# Restoring the Platelet miR-223 by Calpain Inhibition Alleviates the Neointimal Hyperplasia in Diabetes

**DOI:** 10.3389/fphys.2020.00742

**Published:** 2020-07-07

**Authors:** Meiling Su, Shunyang Fan, Zhenwei Ling, Xuejiao Fan, Luoxing Xia, Yingying Liu, Shaoying Li, Yuan Zhang, Zhi Zeng, Wai Ho Tang

**Affiliations:** ^1^Joint Program in Cardiovascular Medicine, Affiliated Guangzhou Women and Children’s Medical Centre, Zhongshan School of Medicine, Sun Yat-sen University, Guangzhou, China; ^2^Institute of Pediatrics, Guangzhou Women and Children’s Medical Centre, Guangzhou Medical University, Guangzhou, China; ^3^Heart Center, The Third Affiliated Hospital of Zhengzhou University, Zhengzhou, China

**Keywords:** diabetes mellitus, platelet, vascular smooth muscle cells, miR-223, calpain, neointimal hyperplasia

## Abstract

Platelet hyperactivity is the hallmark of diabetes, and platelet activation plays a crucial role in diabetic vascular complications. Recent studies have shown that upon activation, platelet-derived miRNAs are incorporated into vascular smooth muscle cells (VSMCs), regulating the phenotypic switch of VSMC. Under diabetes, miRNA deficiency in platelets fails to regulate the VSMC phenotypic switch. Therefore, manipulation of platelet-derived miRNAs expression may provide therapeutic option for diabetic vascular complications. We seek to investigate the effect of calpeptin (calpain inhibitor) on the expression of miRNAs in diabetic platelets, and elucidate the downstream signaling pathway involved in protecting from neointimal formation in diabetic mice with femoral wire injury model. Using human cell and platelet coculture, we demonstrate that diabetic platelet deficient of miR-223 fails to suppress VSMC proliferation, while overexpression of miR-223 in diabetic platelets suppressed the proliferation of VSMC to protect intimal hyperplasia. Mechanistically, miR-223 directly targets the insulin-like growth factor-1 receptor (IGF-1R), which inhibits the phosphorylation of GSK3β and activates the phosphorylation of AMPK, resulting in reduced VSMC dedifferentiation and proliferation. Using a murine model of vascular injury, we show that calpeptin restores the platelet expression of miR-223 in diabetes, and the horizontal transfer of platelet miR-223 into VSMCs inhibits VSMC proliferation in the injured artery by targeting the expression of IGF-1R. Our data present that the platelet-derived miR-223 suppressed VSMC proliferation via the regulation miR-223/IGF-1R/AMPK signaling pathways, and inhibition of calpain alleviates neointimal formation by restoring the expression of miR-223 in diabetic platelet.

## Introduction

Diabetes mellitus (DM) is an important risk factor of intima hyperplasia and vascular restenosis, resulting in diabetic vascular complications (Kornowski et al., 1997; Davi and Patrono, 2007). It is known that platelet hyperreactivity is the hallmark of DM, and the activated platelets (APs) release thromboxane and PDGF, which induce vascular smooth muscle cells (VSMCs) switching from a quiescent contractile phenotype to a highly synthetic and proliferating phenotype upon vascular injury and repair (Millette et al., 2005). Excessive proliferation of VSMCs leads to intimal hyperplasia in DM (Faries et al., 2001). Therefore, the regulation of VSMC phenotypic switch plays a crucial role in maintaining the vascular stability.

Although platelets are anucleate cells, they contain enriched and diverse miRNAs and mRNAs (Landry et al., 2009; Nagalla et al., 2011). We and others have demonstrated that the platelet-derived miRNAs can be transferred and incorporated into recipient cells such as VSMCs, endothelial cells, and macrophages (Risitano et al., 2012; Kirschbaum et al., 2015; Zeng et al., 2019). Our study has also shown that the horizontal transfer of platelet miRNA (miR-143/145 and miR-223) inhibits VSMC dedifferentiation and proliferation, but the altered miRNA expression of diabetic platelets fails to inhibit VSMC dedifferentiation, consequently leading to increased neointimal formation (Zeng et al., 2019). Recent report has shown that Dicer is a calpain substrate, and calpain-dependent cleavage of Dicer reduced the expression of miRNAs in diabetic platelets (Elgheznawy et al., 2015). The altered expression of platelet miRNAs such as miR-223 can be restored by treatment of a calpain inhibitor in diabetic mice (Elgheznawy et al., 2015), suggesting that calpain inhibitor may provide therapeutic adjunct for platelet-associated vascular disease in diabetes.

In the present study, we seek to elucidate the mechanism by which inhibition of calpain regulated VSMC phenotypic switch via upregulating the expression of platelet-derived miRNAs, and the potential application of calpeptin on platelet-associated vascular disease in diabetes. Here we demonstrate that diabetic platelet deficient of miR-223 fails to suppress VSMC proliferation, while overexpression of miR-223 in diabetic platelets suppressed the proliferation of VSMC to protect intimal hyperplasia. Mechanistically, miR-223 directly targets the insulin-like growth factor-1 receptor (IGF-1R), which inhibits the phosphorylation of GSK3β and activates the phosphorylation of AMPK, resulting in reduced VSMC dedifferentiation and proliferation. Using a murine model of vascular injury, we show that calpeptin restores the platelet expression of miR-223 in diabetes, and the horizontal transfer of platelet miR-223 into VSMCs inhibits VSMC proliferation in the injured artery by targeting the expression of IGF-1R. Collectively, our results demonstrate that the platelet-derived miR-223 inhibits VSMC proliferation via miR-223/IGF-1R/AMPK signaling axis, and inhibition of calpain alleviates neointimal formation by restoring the expression of miR-223 in diabetic platelet.

## Materials and Methods

### Platelet Isolation and Purification

Human peripheral blood was obtained from Guangzhou Women and Children’s Medical Center. Informed consent of underage subjects was obtained from their parents. Our study was compiled and approved by the Institutional Review Board of Guangzhou Women and Children’s Medical Center (Human Investigation Committee No. 201944101). Venous blood samples (approximately 4 ml) were drawn from Non-DM and DM subjects from Guangzhou Women and Children’s Medical Center. Venous blood samples (approximately 4 ml) were drawn into blood collection tubes containing 3.8% trisodium citrate (w/v). Platelet-rich plasma (PRP) was centrifuged at 1200 rpm at 25°C for 15 min. PRP were then treated with 100 nM Prostaglandin E1 (PGE1, Sigma) and centrifuged at 2500 rpm for another 5 min. After discarding the supernatant, the platelet pellet was resuspended with 3 ml Hank’s Balanced Salt Solution (HBSS, NaCl 135 mM, MgCl_2_ 1 mM, NaHCO_3_ 12 mM, CaCl_2_ 1 mM, KCl 2.9 mM, Glucose 5 mM, BSA 0.35%, HEPES 10 mM).

For murine platelets, blood was drawn from the left ventricle after anesthesia. Platelet-rich plasma (PRP) was prepared by centrifuging at 1200 rpm for 15 min, and blood coagulation was inhibited by adding the anticoagulant citrate dextrose solution. By centrifuging at 2600 rpm for 5 min, washed platelets were prepared from PRP and were resuspended in HBSS. Platelet concentration was adjusted at 2 × 10^8^ cells/mL using a platelet counter. To prevent platelet activation, the centrifuge temperature was kept at room temperature during the washing step.

### Animals

The male C57BL/6 mice (8 weeks old) were purchased from Guangzhou Medical University. Procedures were approved by the Institutional Animal Care and Use Committee of Guangzhou Medical University (No. SYXK2016-0168). Eight week old mice were injected with 50mg/kg streptozotocin (STZ, Sigma) to induce diabetes for 5 consecutive days. The mice were randomly divided into four groups and were intraperitoneally injected PBS or calpeptin (calpain inhibitor) (30 mg/kg/day) diluted in DMSO in PBS every other day. The mice in the AgomiR group were administered with Agomir-223 200 μl at a concentration of 20 mM via tail vein injection. The calpeptin ware purchased from the SelleckChemicals Co, while the AgomiRs and miRNA mimic from Shanghai GenePharma Co.

### Femoral Artery Wire Injury Model

The endothelial injury in the right femoral artery was done by guidewire abrasion. After anesthetizing the experimental mice, the right thigh was depilated from hair and disinfected with alcohol. The proximal part of the femoral artery and saphenous artery were dissected under the microscope, followed by a temporary stop of blood flow with a silk thread. Then the 0.25 mm diameter guidewire was inserted into the femoral artery from the saphenous artery, inserted and withdrawn 3 times to sufficiently create endothelial wear. The saphenous artery was ligated and the wound was sutured to allow the mice to enter a recovery phase. The contralateral femoral artery was sham-operated without guidewires injured as control. Four weeks after injury, the mice were anesthetized, sacrificed for harvesting the femoral, and fixed in 4% paraformaldehyde. After 30% sucrose dehydration, the arterial tissue was embedded in the Tissue-Tek O.C.T (SAKURA, United States) agent and performed a frozen section.

### Cell Culture

Human coronary artery smooth muscle cells (HCASMC) were purchased from Cell Application at passage 4, cultured in High Glucose DMEM (Gibco, United States) supplemented with 10% fetal bovine serum (Gibco, United States), 100 U/ml penicillin (Gibco, United States), and 100 μg/ml streptomycin (Gibco, United States). The cells were cultured in the incubator with 5% CO_2_ and 37°C. HCASMCs were cocultured with or without resting platelets (RPs) or 0.1 U/ml thrombin activated platelets (APs). After isolation and purification, platelets from healthy subject (HS) and DM patients were counted and added directly to the cultured VSMC (1: 100) for 48 h.

### Transfection of miRNA

HCASMCs were transfected with miR-223 mimic or miR negative control (miR-NC, GenePharma, China) at 100 nM by using Lipofectamine RNAiMAX reagent (Invitrogen, United States). Cells were seeded to be 60–80% confluent before transfection. Lipofectamine reagent and miRNA mimic were diluted in Opti-MEM Medium (Gibco, United States) and mixed for 5 min before added to HCASMCs. Also, HCASMCs were transfected with miR-223 antagomir and miR-NC (Shanghai GenePharma Co) for 24 h, and then VSMCs were cocultured with the purified platelets from HS. After cultured for 48 h, the cells were harvested for subsequent experiments.

### CCK8 Assay

Cell Counting Kit-8 Kits (CCK8; Dojindo, Japan) were used to evaluate cell proliferation. HCASMCs were seeded into a 96-well plate at a concentration of 1.0 × 10^4^ cells/well. Post-transfection 48 h, HCASMCs were incubated with 10 μl CCK8 in 100 μl DMEM for 2 h. The absorbance was detected at 450 nm with a microplate reader (Thermo Fisher Scientific).

### BrdU Incorporation Assay

The HCASMCs were starved in the DMEM without serum and then were transfected with miR-223 mimic and miR-NC as described above. Forty-eight hours after transfection, cell proliferation was determined by using the BrdU Cell Proliferation Assay Kit (Millipore, United States) according to the manufacturer’s protocol. Before measurement, BrdU was added to the cultured cells and incubated for 12 h. After washing with PBS, the cells were fixed and DNA denatured with 4% paraformaldehyde. After fixation, cells were labeled with peroxidase-conjugated BrdU antibody and then incubated with peroxidase substrate. Finally, a stop solution was added to stop the reaction. The absorbance (A_450_–A_550 nm_) representing the cell proliferation was measured on a microplate reader.

### EdU Imaging Assay

Cell proliferation rate was measured with Click-iT EdU Alexa Fluor 488 Imaging Kit (Invitrogen, United States). HCASMCs were seeded into glass-bottom culture dishes at a concentration of 1.0 × 10^4^ cells/ml and transfection experiments were performed as described above. Before termination of cell culture, EdU was added at a final transformation concentration 10 μg/L and incubated with cells at 37°C for 1 h. After incubation, cells were washed with PBS, fixed with 3.7% paraformaldehyde for 15 min, and permeabilized with 0.5% Triton^®^X-100 for 20 min. The Click-iT^®^ reaction cocktail prepared 15 min in advance was added to the notch of each well and incubated at room temperature for 30 min, protected from light. After washing, DNA staining was performed using Hoechst 33342 solution at a concentration of 1: 2000. The EdU positive cells were observed and captured under the inverted fluorescence microscope (Leica), and the cell count was calculated by using Image J.

### Luciferase Reporter Assay

IGF-1R 3′-UTR plasmid was obtained from Biolink Technology Co. The HEK-293 cells were plated in a 24-well plate in a concentration of 50%. After 24 h plated, the 1μg plasmid and 50 nM miR-223 mimic and miR-NC were transfected by using Lipofectamine RNAiMAX reagent (Invitrogen, United States). Luciferase reporter analyses were performed according to the manufacturer’s instructions after 48 h of transfection by using Dual Luciferase Assay Kit (Promega, United States). The Firefly and Renilla fluorescence values were detected by the microplate infinite M1000 (Tecan) and the firefly fluorescence normalized by Renilla was served as luciferase activity.

### HE Staining

The frozen sections of the tissues were dried at room temperature for 30 min and placed in PBS for 10 min. According to the following sequence, the tissues were placed in hematoxylin for 1 min, hydrochloric acid alcohol for 2,3 sec, and then moved to eosin for 8–10 sec. Finally, the tissues were rinsed in running water and then mounted with neutral gum.

### Immunofluorescence

Cells and tissues were fixed and permeabilized by 4% paraformaldehyde (Servicebio, China) and 0.2% Triton-100X (Solarbio, China) in PBS, respectively; staining was with antibodies and their corresponding secondary antibodies. Primary antibodies used include IGF-1R (1:100, ab182408, Abcam, United States) and α-smooth muscle actin (Acta2; 1:100, ab119952, Abcam, United States). After 24 h, sections were incubated with the Alexa Fluor 594-conjugated anti-rabbit IgG secondary antibody and Alexa Fluor 488-conjugated anti-goat IgG secondary antibody (Thermo Fisher Scientific) (1:200) for 60 min and stained with the chromatin-specific dye Hoechst for 5 min at RT. The confocal images were taken with a Leica SP8 (Leica) confocal microscope.

### Western Blot Analysis

The cells were harvested with RIPA lysate containing protease inhibitors or phosphatase inhibitor cocktail. Extract protein by centrifugation at 4°C at 12,000 rpm and heated at 95°C with loading buffer. The protein samples were resolved in 12% SDS-PAGE gels and transferred to PVDF membranes (Millipore, United States). PVDF membranes were incubated with 5% skim milk powder (Biofroxx, Germany) for 2 h and then incubated with primary antibodies used including: IGF-1R (1:1000, ab182408, Abcam, United States); ACTA2 (1:2000, ab119952, Abcam, United States); calponin (1:2000, CNN1; ab46794, Abcam, United States); transgelin (TAGLAN; 1:2000, ab14106, Abcam, United States); KLF4 (1:2000, 101508, GenetTex, United States); KLF5 (1:2000, ab137676, Abcam, United States); osteopontin (1:2000, OPN; ab91655, Abcam, United States); Akt (1:1000, 9272S, CST, United States); Phospho-Akt (1:500, Ser473) (4060S, CST, United States); GSK3β (1:1000, 9315S, CST, United States); Phospho-GSK3β (Ser9) (1:500, 9336S, CST, United States); Src (1:1000, MAB3389, R&D, United States); Phospho-Src (Y419) (1:500, AF2685, R&D, United States); JNK (1:1000, 9252S, CST, United States); Phospho-JNK (Thr172) (1:500, 4668S, CST, United States); AMPK (1:1000, 2532S, CST, United States); Phospho-AMPK (Thr183/Tyr185) (1:500, 2535S, CST, United States); Acetyl-CoA Carboxylase (1:1000, ACC; 3662S, CST, United States); Phospho-ACC (Ser79) (1:500, 3661S, CST, United States); p21 (1:1000, 2847, CST, United States); P27 (1:1000, 3688, CST, United States); GAPDH (1:3000, ab125247, Abcam, United States); α-tubulin (1:3000, ab52866, Abcam, United States); α-actinin (1:3000, ab18061, Abcam, United States). The next day, the PVDF membranes were incubated with a secondary antibody (1:2000) dissolved in 5% nonfat milk after three times of washing with TBST. The specific band for the target protein was detected by ChemiDoc XRS+ system, and the band intensities were calculated using the Image Lab software.

### Proteomic Phosphokinase Array

The cell lysate was extracted from the cells after the treatment as described above, and the protein was quantified with a bicinchoninic acid kit. According to the manufacturer’s instructions, after blocking with Array Buffer 1, the phosphokinase array membrane was incubated with diluted cell lysate overnight. After washing three times with Wash Buffer to remove unbound protein, the array was incubated with a cocktail of biotinylated detection antibodies for 2 h at room temperature. Streptavidin-HRP and chemiluminescent detection reagents were applied, and the signal was detected by ChemiDoc XRS+ system. Refer to the coordinates of analytes and controls provided in the appendix of the kit instructions, the signal at each capture spot corresponding to the amount of phosphorylated protein bound was analyzed.

### Extraction and Purification of miRNA and qRT-PCR

Total RNA purification kit (Axygen, United States) was used to extract and purify miRNA including miRNA from cells, and purification of miRNA were reverse transcribed with miRNeasy Serum/Plasma kit (Qiagen, Germany) and U6 snRNA Normalization RT-PCR Quantitation Kit (GenePharma). After normalizing the nucleic acid concentration by quantification, reverse transcription PCR was performed to obtain stable cDNA by using the PrimeScript RT reagent Kit (Takara, Japan). Transcript levels of the target genes were analyzed by qPCR using the 2^–ΔΔCt^ method. The primer sequences were listed in Supplementary Table 1.

### Statistical Analysis

All data are presented as mean ± SD. All analysis was performed using Graph Pad Prism v.5.0 (GraphPad, La Jolla, CA, United States). Comparisons between two groups were analyzed using unpaired nonparametric Student’s *t*-test. Comparisons between more than two groups were analyzed using one-way ANOVA analysis with the Dunnett’s multiple correction. *P* < 0.05 was considered statistically significant.

## Results

### MiR-223 Deficiency in Diabetic Platelet Failed to Suppress VSMC Proliferation

**FIGURE 1:**
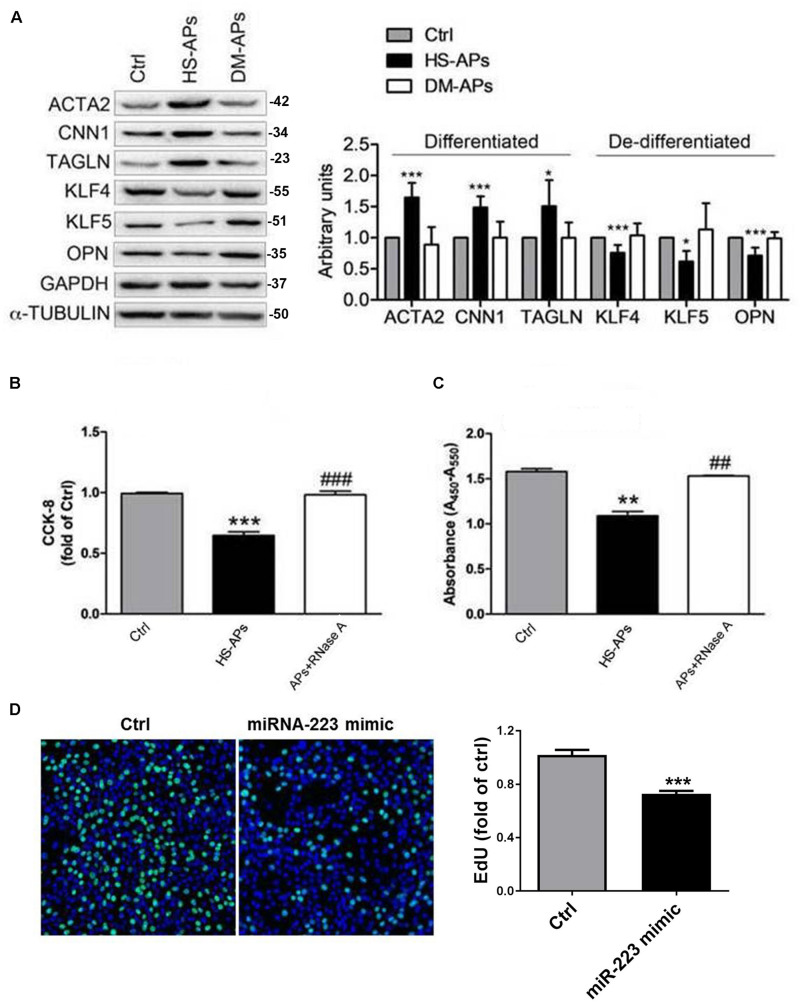
MiR-223 deficiency in diabetic platelet fails to suppress VSMC proliferation. Activated platelets (APs) from healthy subject (HS) and DM patients were cocultured with human VSMCs for 48 h. **(A)** Expression of differentiation markers ACTA2, CNN1, and TAGLN and dedifferentiation markers OPN, MYH10, KLF4, and KLF5 in control (Ctrl) VSMCs and APs cocultured with VSMCs (HS-APs, DM-APs) was detected with western blot analysis. **(B,C)** The rate of proliferation was determined by CCK8 assay and Brdu assay in VSMCs treated with APs pretreated by RNase A (degrades the RNA). Data are presented as mean ± SD (*n* = 6). Comparisons between more than two groups were analyzed using one-way ANOVA analysis with the Dunnett’s multiple correction. *P < 0.05, **P < 0.01, ***P < 0.001 vs Ctrl (VSMCs without platelets); ^#^*P* < 0.05, ^##^*P* < 0.01, ^###^*P* < 0.001 vs HS-APs. **(D)** The rate of proliferation was determined by EdU incorporation assay in VSMCs transfected with miR-223 mimic. Data are presented as mean ± SD (*n* = 6). Comparisons between two groups were analyzed using unpaired nonparametric Student’s *t*-test.*P < 0.05 vs Ctrl (miR-NC).

Consistent to our previous (Zeng et al., 2019), VSMCs cocultured with DM-APs showed downregulation of VSMCs differentiation markers α-SMA (ACTA2), Calponin (CNN1), and SM22α (TAGLN) compared to HS-APs group (Figure 1A). Also, the expression of VSMCs dedifferentiation markers Osteopontin (OPN), MYH10, KLF4, and KLF5 was significantly increased in VSMCs cocultured with DM-APs (Figure 1A). Pretreatment with miR-223 antagomir, we found that the SMCs differentiation markers ACTA2 and CNN1 of VSMCs cocultured with HS-APs treated by miR-223 antagomir was significantly inhibited than VSMCs cocultured with HS-APs. Also, the expression of VSMCs dedifferentiation markers KLF4 was significantly increased in VSMCs cocultured with HS-APs treated by miR-223 antagomir (Supplementary Figure S1). Pretreatment with RNase A (degrades the RNA), we found that the proliferation rate of VSMCs cocultured with APs treated by RNase A was significantly higher than VSMCs cocultured with APs (Figure 1B). Also, overexpression of miR-223 significantly suppressed VSMC proliferation (Figures 1C,D). These results suggested that platelet-derived miR-223 was transferred and incorporated into VSMCs, inhibiting VSMC proliferation. Deficiency in diabetic platelet fails to suppress VSMC proliferation.

### MiR-223 Directly Targets the IGF-1R, Which Inhibits the Phosphorylation of Akt and GSK3β

**FIGURE 2:**
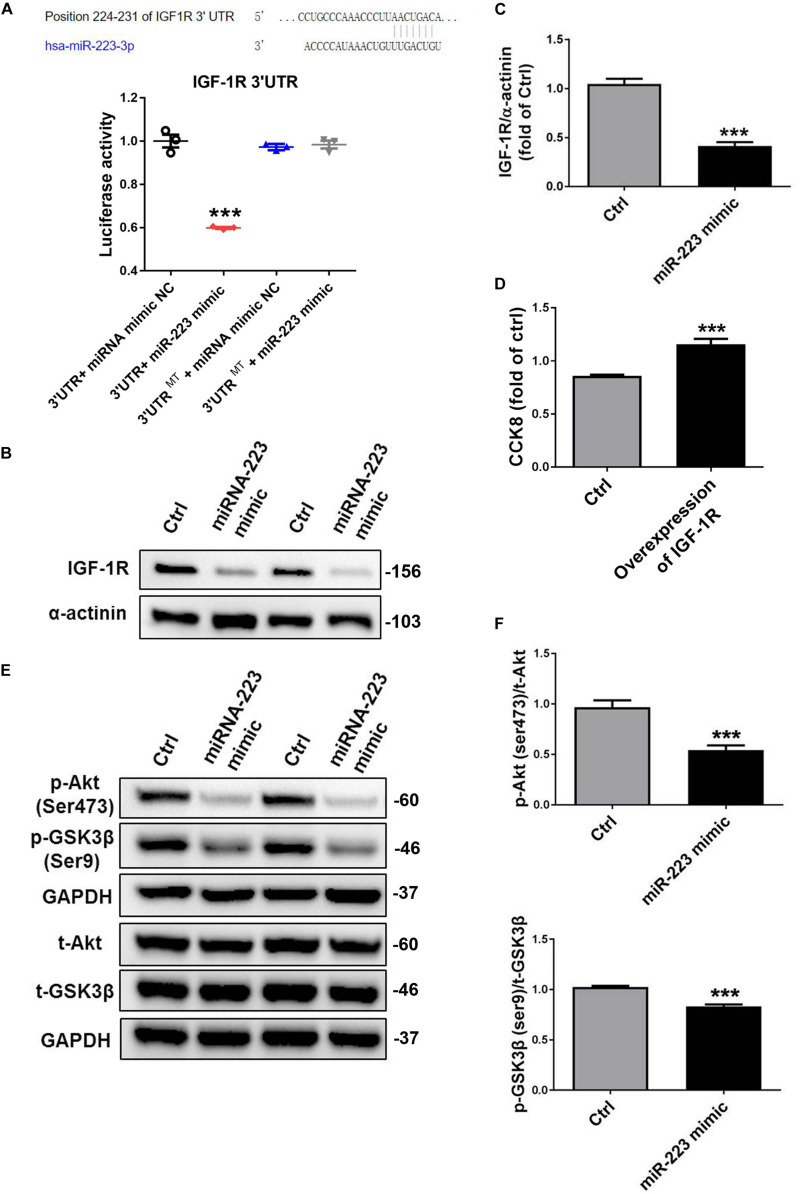
MiR-223 directly targets the IGF-1R, which inhibits the phosphorylation of Akt and GSK3β. **(A)** Bioinformatics analysis identified a binding site for miR-223 in the 3′-UTR of IGF-1R. Luciferase activity assay in VSMCs following introduction of IGF-1R 3′ UTR or mutant 3′ UTR (mut) with or without miR-223 mimic (*n* = 3). VSMCs were transfected with miR-223 mimic for 48 h. **(B,C)** The expression of IGF-1R in VSMC was detected with western blot analysis. **(D)** The rate of proliferation was determined by CCK8 assay in VSMCs transfected with overexpression of IGF-1R. **(E,F)** The phosphorylation of Akt and GSK3β in VSMC was determined with western blot analysis. Data are presented as mean ± SD (*n* = 6). Comparisons between two groups were analyzed using unpaired nonparametric Student’s *t*-test. *P < 0.05, **P < 0.01, ***P < 0.001 vs Ctrl (miR-NC).

Bioinformatics analysis identified a binding site for miR-223 in the 3′-UTR of IGF-1R. When we cloned IGF-1R 3′ UTR into luciferase report, we found that a miR-223 mimic significantly inhibited the luciferase activity; however, the inhibition of luciferase activity was abrogated by mutated control group (Figure 2A). To identify whether miR-223 could bind to IGF-1R and inhibited the expression, we performed that VSMC was treated with a miR-223 mimic. As expected, the expression of IGF-1R in VSMC was significantly decreased by overexpression of miR-223 compared to control group. The data suggest miR-223 can directly target the 3′-UTR of IGF-1R and inhibit its expression in VSMC (Figures 2A–C). Also, we performed overexpression of IGF-1R in VSMCs. We found that VSMC proliferation was significantly increased in overexpression of IGF-1R of VSMCs compared with control group using CCK8 (Figure 2D). The binding of IGF-1 to its receptor (IGF-1R) can activate the cascade signals including IRS family, thereby activating phosphatidyl inositol 3-kinase (PI3K) and Akt signaling pathway (Delafontaine et al., 2004). Thus we determined the downstream signal pathway involved in inhibiting VSMC proliferation treated with a miR-223 mimic. Figures 2E,F showed that the phosphorylation of Akt and GSK3β was inhibited by the miR-223 mimic than that of control group. Our data demonstrate that miR-223 directly targets IGF-1R in VSMC, which inhibits the phosphorylation of Akt and GSK3β, resulting in reduced VSMC dedifferentiation and proliferation.

### APs Activate the AMPKα Signal Pathway to Suppress VSMC Proliferation

**FIGURE 3:**
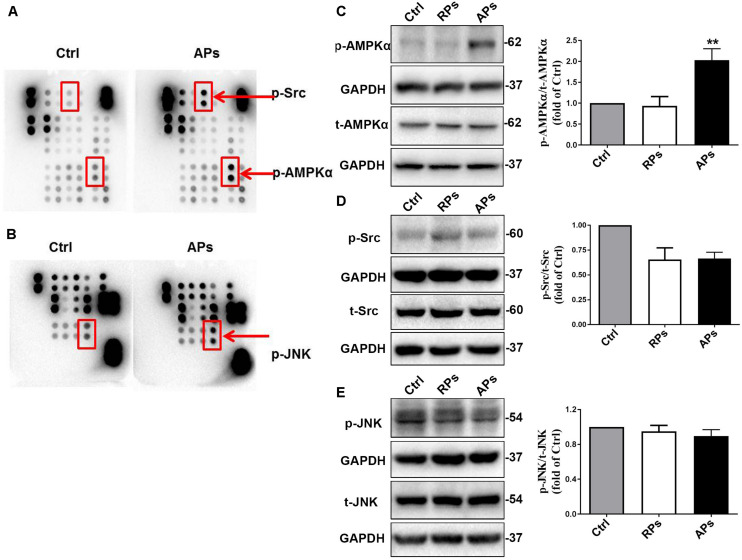
APs activate the AMPKα signal pathway to suppress VSMC proliferation. **(A,B)** The phosphorylation of AMPKα, Src and Jun were assessed by proteomics analysis of phosphokinase arrays in control (Ctrl) VSMCs and APs cocultured with VSMCs. The phosphorylation of **(C)** AMPKα, **(D)** Src, and **(E)** Jnk was determined with western blot analysis in control (Ctrl) VSMCs, RPs, and APs cocultured with VSMCs. Data are presented as mean ± SD (*n* = 6). Comparisons between more than two groups were analyzed using one-way ANOVA analysis with the Dunnett’s multiple correction. **P < 0.01 vs Ctrl (VSMCs without platelets).

To further determine the downstream signal pathway of Akt/GSK3β involved in inhibiting VSMC proliferation, we determined that the phosphorylation of AMPKα, Src and Jnk were assessed by proteomics analysis of phosphokinase arrays in control (Ctrl) VSMCs and APs cocultured with VSMCs. As shown in Figures 3A,B, the phosphorylation of AMPKα, Src and Jnk in VSMCs treated with APs were significantly increased compared to the control group. Using western blot analysis, we further confirmed that the phosphorylation of AMPKα in VSMCs treated with APs was significantly upregulated compared to the control group (Figure 3C). However, we found that the phosphorylation of Src and Jnk did not change in VSMCs cocultured with resting platelets (RPs) or APs (Figures 3D,E). These results suggest that APs activate the AMPKα signal pathway to suppress VSMC proliferation.

**FIGURE 4:**
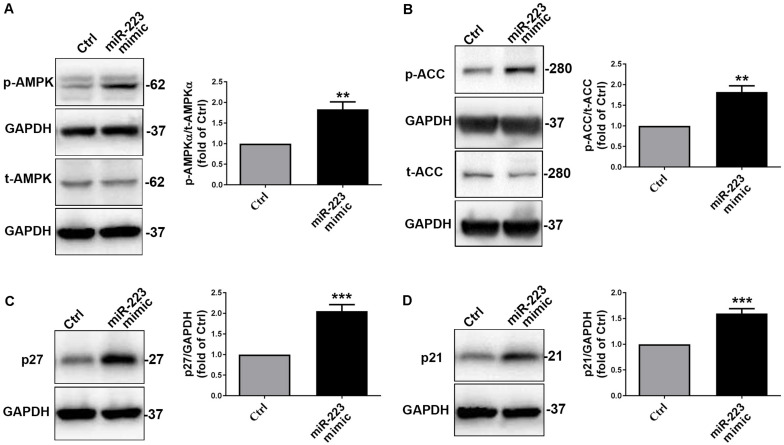
The platelet-derived miR-223 suppressed VSMC proliferation via the regulation AMPK signaling pathways. VSMCs were transfected with miR-223 mimic for 48 h. **(A,B)** The phosphorylation of AMPKα and ACC were assessed by western blot analysis in VSMCs transfected with miR-223 mimic. **(C,D)** The expression of cell cycle marks including p27 and p21 was determined with western blot analysis in VSMCs transfected with miR-223 mimic. Data are presented as mean ± SD (*n* = 6). Comparisons between two groups were analyzed using unpaired nonparametric Student’s *t*-test. *P < 0.05, **P < 0.01, ***P < 0.001 vs Ctrl (miR-NC).

To further investigate whether platelet-derived miR-223 suppressed VSMC proliferation through AMPKα signal pathway, we treated with a miR-223 mimic or miR negative control (Ctrl) in VSMCs. Similarly, we found that the phosphorylation of AMPKα in VSMCs with treatment of a miR-223 mimic was significantly increased compared to that of the Ctrl group (Figure 4A). As expected, we found that the phosphorylation of AMPKα in VSMCs cocultured with DM-APs was significantly inhibited compared to that in VSMCs with treatment of HS-APs (Supplementary Figure S2). We found that the phosphorylation of acetyl-CoA Carboxylase (ACC), the substrate of AMPKα, was significantly higher in VSMCs treated with a miR-223 mimic than Ctrl group (Figure 4B). We also found that treatment with miR-223 mimic significantly increased the expression of p27 and p21 (Figures 4C,D), suggesting that VSMC proliferation was suppressed by miR-223. Taken together, these results indicated that the platelet-derived miR-223 suppressed VSMC proliferation via the regulation of miR-223/IGF-1R/AMPK signaling pathways.

### Inhibition of Calpain Restored the Expression of miR-223 in Diabetic Platelets

**FIGURE 5:**
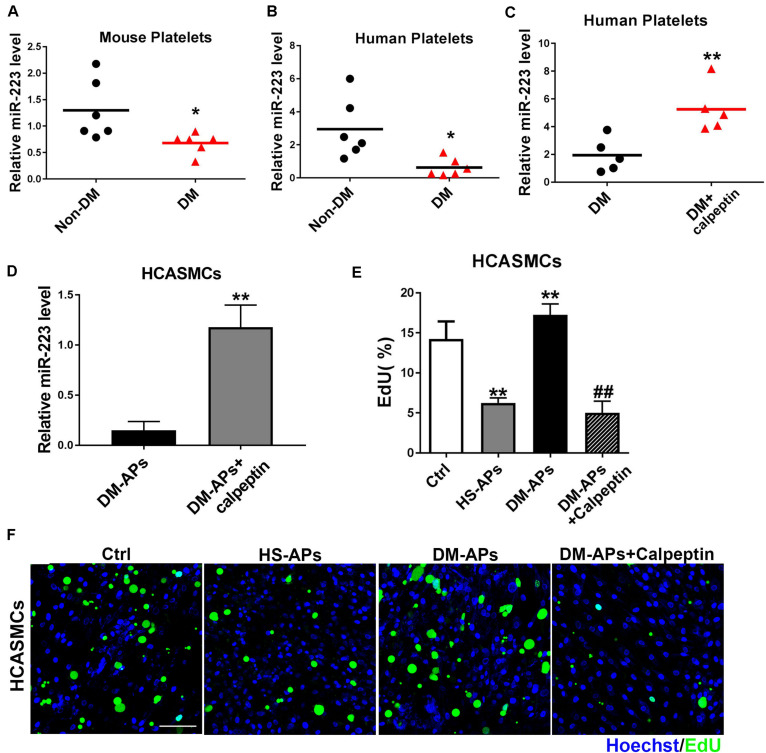
Treatment of a calpeptin restored the expression of miR-223 in diabetic platelets. **(A,B)** The miR-223 level in mouse and human platelets was detected by qRT-PCR assays. Data are presented as mean ± SD (*n* = 6). Comparisons between two groups were analyzed using unpaired nonparametric Student’s *t*-test. *P < 0.05 vs Non-DM. **(C,D)** Diabetic platelets were treated with a calpeptin for 24 h in vitro. The expression of miR-223 in human platelets and HCASMCs was detected by qRT-PCR assays. Data are presented as mean ± SD (*n* = 6). Comparisons between two groups were analyzed using unpaired nonparametric Student’s *t*-test. **P < 0.01 vs DM. **(E,F)** The rate of proliferation was determined by EdU incorporation assay in VSMCs treated with calpeptin. Data are presented as mean ± SD (*n* = 6). Comparisons between more than two groups were analyzed using one-way ANOVA analysis with the Dunnett’s multiple correction. **P < 0.01 vs Ctrl (VSMCs without platelets); ^##^*P* < 0.01 vs DM-APs.

Our previous study has demonstrated a significant upregulation of miRNAs including miR-223, miR-143, and miR-145 in VSMCs cocultured with APs compared to VSMCs group. As shown in Figures 5A,B, the expression of miR-223 was downregulated in mouse and human diabetic platelets compared with non-DM group. We found that miR-223 level was significantly higher in diabetic platelets treated with a calpeptin (calpain inhibitor) than that of diabetic platelets (Figures 5C,D), suggesting that inhibition of calpain restores the expression of miR-223 in diabetic platelets. After restoring the expression of miR-223 in diabetic platelets and then coculturing with HCASMCs, we found the inhibition of Calpain restored the expression of miR-223 in diabetic platelets suppressed the proliferation of HCASMCs (Figures 5E,F). These data demonstrate that calpeptin restores the expression of miR-223 in diabetic platelets and transferred more platelet-derived miR-223 into VSMC, thereby suppressing VSMC proliferation in vitro.

### Inhibition of Calpain Alleviates Neointimal Formation by Restoring the Expression of miR-223 in Diabetic Platelet

**FIGURE 6:**
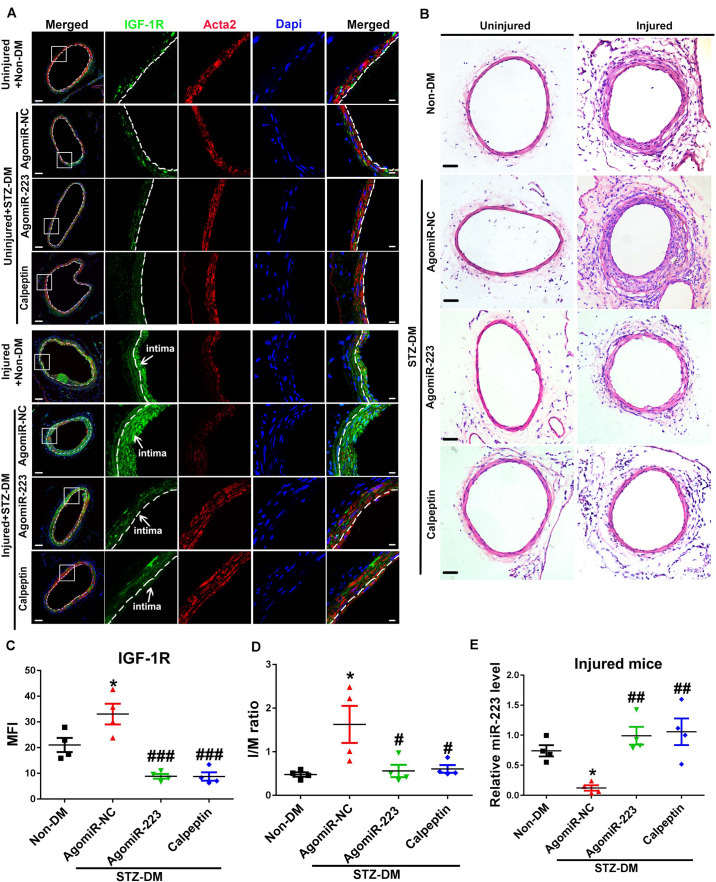
Inhibition of calpain alleviates neointima formation by restoring the expression of miR-223 in diabetic platelet. **(A)** Immunofluorescence analysis of IGF-1R in uninjured or injured femoral arteries from Non-DM mice, and STZ-DM mice treated with AgomiR-NC or AgomiR-223 or calpeptin at 4 weeks after wire injury (*n* = 4). Green, IGF-1R; red, Acta2 blue; Dapi nuclear staining in VSMCs. Scale bars: 20 μm. **(B)** H&E staining of serial cross sections of femoral arteries from Non-DM mice, and STZ-DM mice treated with AgomiR-NC or AgomiR-223 or calpeptin at 4 weeks after wire injury (*n* = 4). Scale bars: 50 μm. **(C)** Quantification of IGF-1R expression in VSMCs in injured DM mice. **(D)** The intima size (I/M ration) in the injured femoral arterial sections has been quantified. **(E)** The miR-223 level in injured DM mice was detected by qRT-PCR assays. Data are presented as mean ± SD (*n* = 4). Comparisons between more than two groups were analyzed using one-way ANOVA analysis with the Dunnett’s multiple correction. *P < 0.05 vs non-DM; ^#^*P* < 0.05, ^##^*P* < 0.01, ^###^*P* < 0.001 vs STZ-DM mice treated with AgomiR-NC.

Calpeptin is a specific pharmacological inhibitor for calpain activation, and administration of calpeptin restored the expression of miR-223 in diabetic murine platelet (Elgheznawy et al., 2015). To restore the downregulated miR-223 in diabetic platelet, we used streptozotocin induced (STZ-DM) mice and rescued the condition with a calpeptin and AgomiR-223 by femoral wire injury. Four weeks after injury, we found the expression of IGF-1R in uninjured vessels was reduced, while the expression of IGF-1R was upregulated in injured vessels of STZ-DM mice treated with miR-NC (miR negative control) (Figures 6A,C). Treatment with AgomiR-223 and calpeptin significantly suppressed the expression of IGF-1R in STZ-DM mice (Figure 6A). The results suggest that calpeptin and AgomiR-223 restored the expression of miR-223 in diabetic platelets to suppress VSMC proliferation via targeting IGF-1R in vivo. To verify the effect of calpeptin and AgomiR-223 on neointimal formation, the intimal to media (I/M ration) was detected in STZ-DM and Non-DM mice. As shown in Figures 6B,D, the neointimal hyperplasia and intimal size were increased in STZ-DM mice after 4 weeks of femoral arterial injury; however, the effect was rescued by treatment with calpeptin and AgomiR-223. We found that the expression of miR-223 was significantly downregulated in diabetic platelets of injured mice compared with non-DM injured group. However, the expression of miR-223 was restored by treatment with calpeptin and AgomiR-223, suggesting that inhibition of calpain restores the expression of miR-223 in injured DM mice (Figure 6E).Collectively, these data demonstrate that calpeptin restores the downregulated miR-223 in diabetic platelets and transferred more platelet-derived miR-223 into VSMC, thereby suppressing VSMC proliferation through targeting IGF-1R in the injured vessel, resulting in alleviation of neointimal formation in diabetes.

**FIGURE 7:**
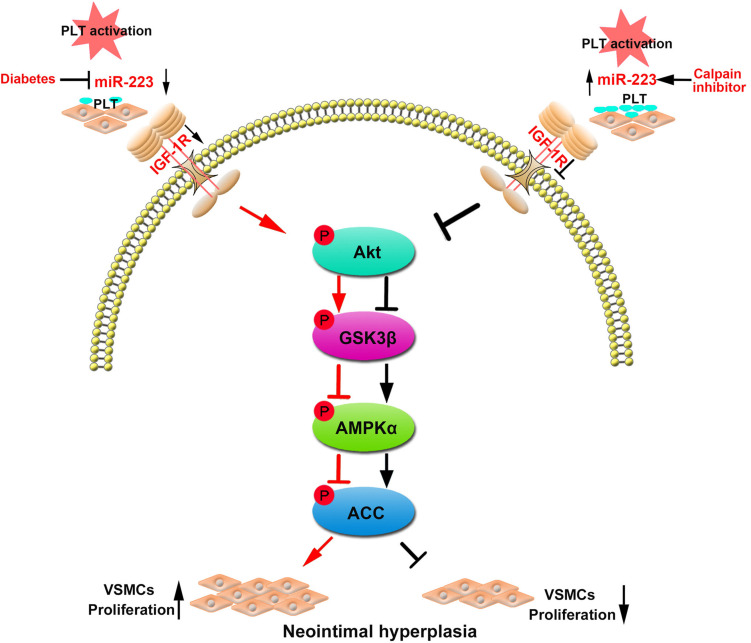
Restoring the platelet miR-223 by calpeptin alleviates the neointimal hyperplasia in diabetes through miR-223/IGF-1R/AMPK signaling pathway. MiR-223 deficiency in diabetic platelet failed to suppress VSMC proliferation during vascular injury. The platelet-derived miR-223 suppressed VSMC proliferation via the regulation IGF-1R/AMPK signaling pathways. Interestingly, treating with a calpeptin restores the downregulated expression of miR-223 in diabetic platelets to suppress VSMC proliferation via targeting IGF-1R and plays a protective role in diabetic neointimal hyperplasia.

In summary, our data manifested that the platelet-derived miR-223 suppressed VSMC proliferation via the regulation IGF-1R/AMPK signaling pathway. However, treatment with calpeptin (calpain inhibitor) restored the expression of miR-223 in diabetic platelets, which was then transferred into VSMCs and inhibited VSMC proliferation at the site of vascular injury. Thus calpeptin may serve as therapeutic adjunct for playing a protective role in diabetic neointimal hyperplasia (Figure 7).

## Discussion

In the present study, we demonstrate that diabetic platelet deficient of miR-223 fails to suppress VSMC phenotypic switch. The incorporated platelet miR-223 directly targets the insulin-like growth factor-1 receptor (IGF-1R) in VSMCs, which inhibits the phosphorylation of GSK3β and AMPK, resulting in reduced VSMC dedifferentiation and proliferation. In murine model of vascular injury, we show that calpeptin restores the platelet expression of miR-223 in diabetes, and the horizontal transfer of platelet miR-223 into VSMCs inhibits VSMC proliferation in the injured artery by targeting the expression of IGF-1R. Collectively, our results demonstrate that inhibition of calpain regulated VSMC phenotypic switch via miR-223/IGF-1R/AMPK signaling axis, supporting the potential application of calpeptin on platelet-associated vascular disease in diabetes.

Diabetes mellitus is an important risk factor of intimal hyperplasia and restenosis (Davi and Patrono, 2007). The phenotypic switch of VSMCs plays a crucial role in the onset of intimal hyperplasia in DM. The process of VSMC phenotypic switch (dedifferentiation and proliferation) was regulated by cytokines including insulin-like growth factor-1 (IGF-1), platelet-derived growth factor (PDGF), and epidermal growth factor (EGF) (Delafontaine and Lou, 1993). Previous studies have reported miR-223 inhibited VSMC proliferation via target ITGB3 or RhoB/MLC2 in VSMCs (Zeng et al., 2016; Liu et al., 2019). Recently, our previous studies have demonstrated PDGFRβ is a key target of platelet-derived miR-223, which is incorporated into VSMCs and suppressed VSMC proliferation (Zeng et al., 2019). Previous studies have demonstrated treating with IGF-1 antibody inhibited VSMC proliferation, which was induced by angiotensin and thrombin (Delafontaine et al., 2004; Ma et al., 2006), suggesting that IGF-1 also plays a crucial role in VSMC proliferation (Jones and Kazlauskas, 2001). The binding of IGF-1 to its receptor (IGF-1R) can activate the cascade signals including IRS family, thereby activating phosphatidyl inositol 3-kinase (PI3K) and Akt signaling pathway (Saltiel and Kahn, 2001; Delafontaine et al., 2004). In the present study, we identified that IGF-1R was the direct target of miR-223, and platelet-derived miR-223 was incorporated into VSMCs thereby inhibiting IGF-1R, leading to the phosphorylation of Akt and GSK3β. Report showed PI3K-Akt signaling pathway could suppress glycogen synthase kinase 3 (GSK3) activity, leading to inhibit the function of AMPK via interactions with AMPKβ (Fukushima et al., 2012; Suzuki et al., 2013). It has been reported that activated phosphorylation of AMPKα to inhibit cells proliferation (Igata et al., 2005; Lee et al., 2015). Our phosphoproteomics array results showed that the incorporated platelet miR-223 significantly increased the phosphorylation of AMPKα and its substrate ACC in VSMCs cocultured with activated platelets. Therefore, platelet-derived miR-223 after incorporated into VSMCs at least in part suppress VSMC proliferation via targeting IGF-1R and activating AMPK to reduce neointimal hyperplasia in DM.

In diabetic platelets, the reduction of miRNAs leads to the upregulation of protein expression such as β1 integrin, resulting in increased platelet aggregation (Elgheznawy et al., 2015). Calpain family is intracellular Ca^2+^-activated cysteine proteases, which involved in cellular signaling, cell growth (Salimi et al., 2018), apoptosis (Storr et al., 2011), and platelet activation in DM (Elgheznawy et al., 2015). Increasing evidence has demonstrated calpain activation involved in cardiovascular complication of DM such as increased vascular permeability (Scalia et al., 2007), atherosclerosis (Miyazaki et al., 2011), and heart failure (Letavernier et al., 2012). The activation of calpain in DM is closely associated with the loss of platelet dicer, resulting in the significant dysregulation of platelet miRNA levels. Calpeptin is a specific pharmacological inhibitor for calpain activation, and administration of calpeptin restored the expression of miR-223 in diabetic murine platelet (Elgheznawy et al., 2015). Moreover, calpeptin was found to be an effective therapeutic drug for preventing endothelial dysfunction of mesenteric arteries in DM (Randriamboavonjy et al., 2017). Consistent with these studies, our results showed that the expression of miR-223 in diabetic platelets was restored by treatment of calpeptin. Thus, we employed calpeptin to treat STZ-DM murine model of vascular injury. We also found that calpeptin has inhibited the activative effect calpain on neointima hyperplasia in STZ-DM murine model of vascular injury. Both treatment with AgomiR-223 and calpeptin reduced neointimal hyperplasia and intimal size. At the site of injury, the increased IGF-1R expression in VSMCs was attenuated by treatment with AgomiR-223 and calpeptin. These results suggest that calpeptin may serve as therapeutic adjunct for intimal hyperplasia via the upregulation of platelet miR-223 in diabetes.

In summary, we provide evidence that the horizontal transfer of platelet miR-223 suppresses VSMC proliferation via targeting IGF-1R and activating AMPK. However, in diabetes, calpain activation contributes to the reduced expression of dicer, resulting in the deficiency of platelet miR-223. Treatment with calpeptin restored the expression of miR-223 in diabetic platelets, which was then transferred into VSMCs and inhibited VSMC proliferation at the site of vascular injury. Therefore, calpeptin may serve as therapeutic adjunct for platelet and VSMC associated vascular diseases in DM.

## Data Availability Statement

The datasets generated for this study are available on request to the corresponding author.

## Ethics Statement

The studies involving human participants were reviewed and approved by Institutional Review Board of Guangzhou Women and Children’s Medical Center (Human Investigation Committee No. 2018022605). Written informed consent to participate in this study was provided by the participants’ legal guardian/next of kin. The animal study was reviewed and approved by Institutional Animal Care and Use Committee of Guangzhou Medical University (No. SYXK2016-0168).

## Author Contributions

MS and SF performed the experimental design, the majority of experiments, analyzed majority of the data, and drafted manuscript. ZL and LX performed part of experiments and the data analyses. XF, YL, YZ, and SL performed animal experiments and image analyses. ZZ and WT directed all aspects of the experiments, the data analyses, manuscript editing and review. All authors contributed to the article and approved the submitted version.

## Conflict of Interest

The authors declare that the research was conducted in the absence of any commercial or financial relationships that could be construed as a potential conflict of interest.
