# Supplementary material for: Restoring the Platelet miR-223 by Calpain Inhibition Alleviates the Neointimal Hyperplasia in Diabetes
Source: Front Physiol. 2020 Jul 7;11:742. doi: 10.3389/fphys.2020.00742 (PMC7359912; doi:10.3389/fphys.2020.00742)
Supplement: Supplementary file 1 [file Data_Sheet_1.docx]

Supplementary Material

# Supplementary Figures


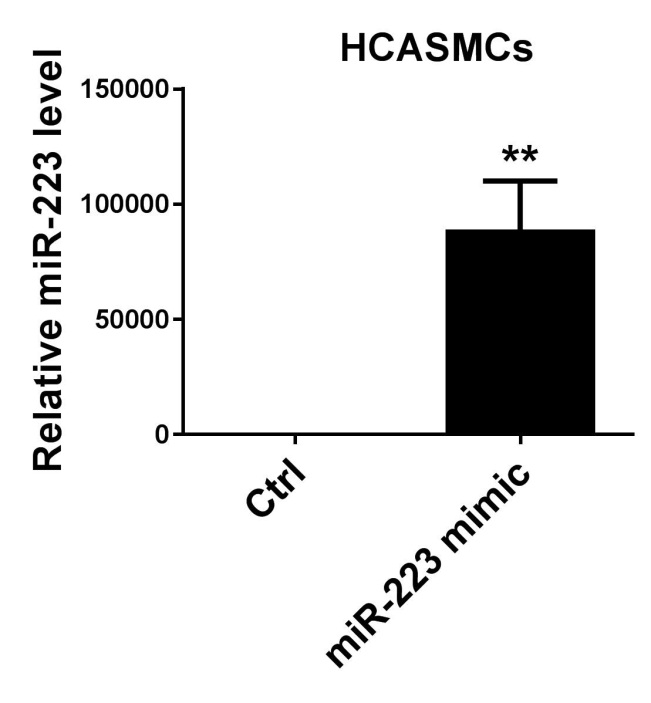


**Figure. S1. The level of miR-223 in HCASMCs after transfection.** HCASMCs were transfected with miR-223 mimic for 48h, the expression of miR-223 were detected by RT-PCR. Data are presented as mean ± SD (n=4). Comparisons between two groups were analyzed using unpaired nonparametric Student’s t-test. **P < 0.01 vs. Ctrl with treatment of miR-NC.

**
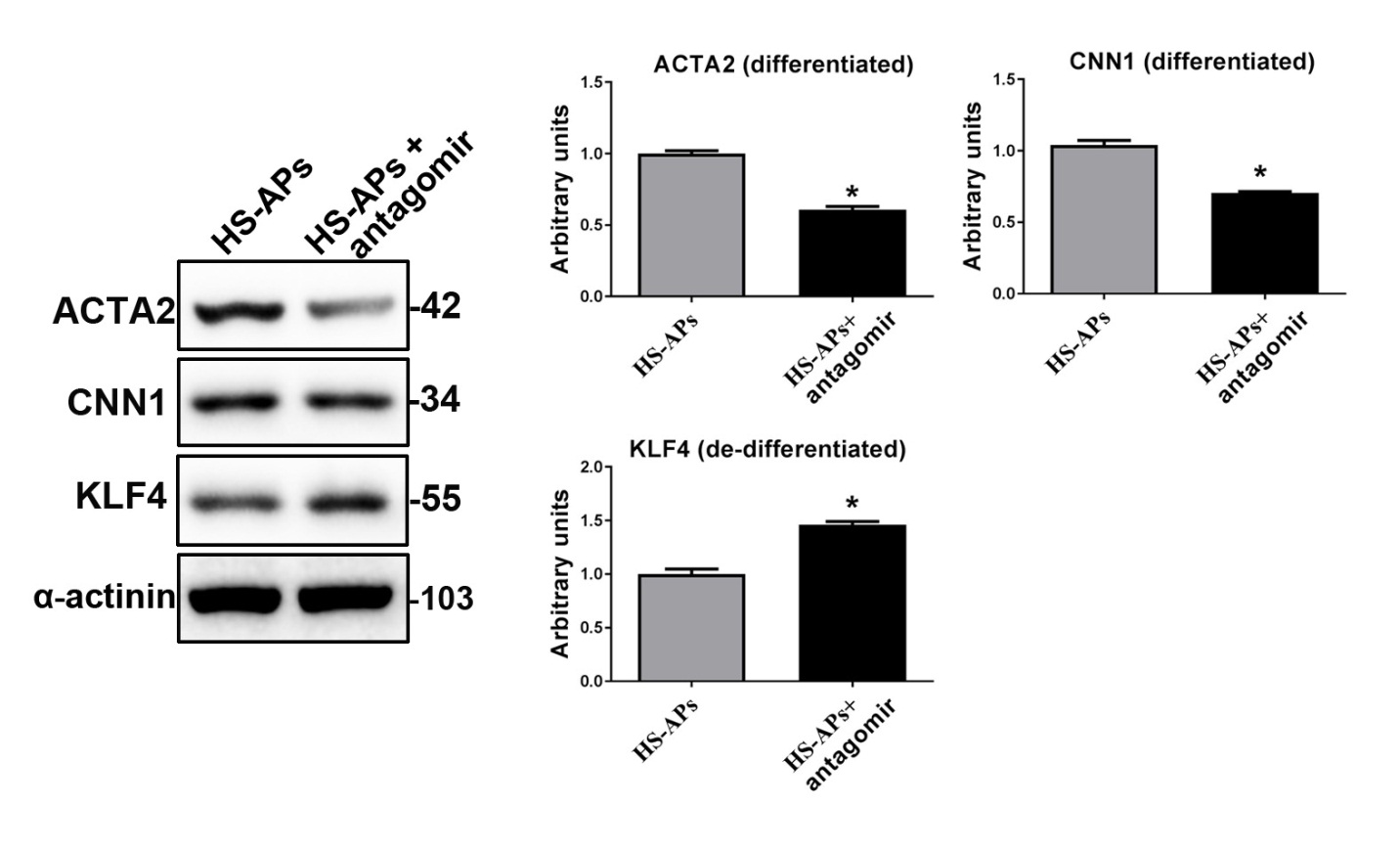
**

**Figure. S2. MiR-223 deficiency in HS platelet treated with miR-223 antagomir fails to suppress VSMC proliferation.** HCASMCs were transfected with miR-223 antagomir for 24h, and then VSMCs were cocultured with the purified platelets from HS., Expression of differentiation markers ACTA2 and CNN1 and dedifferentiation markersKLF4 in VSMCs (HS-APs and HS-APs+ antagomir ) was detected with western blot analysis. Data are presented as mean ± SD (n=3). Comparisons between two groups were analyzed using unpaired nonparametric Student’s t-test. *P < 0.05 vs. VSMCs with treatment of HS-APs.


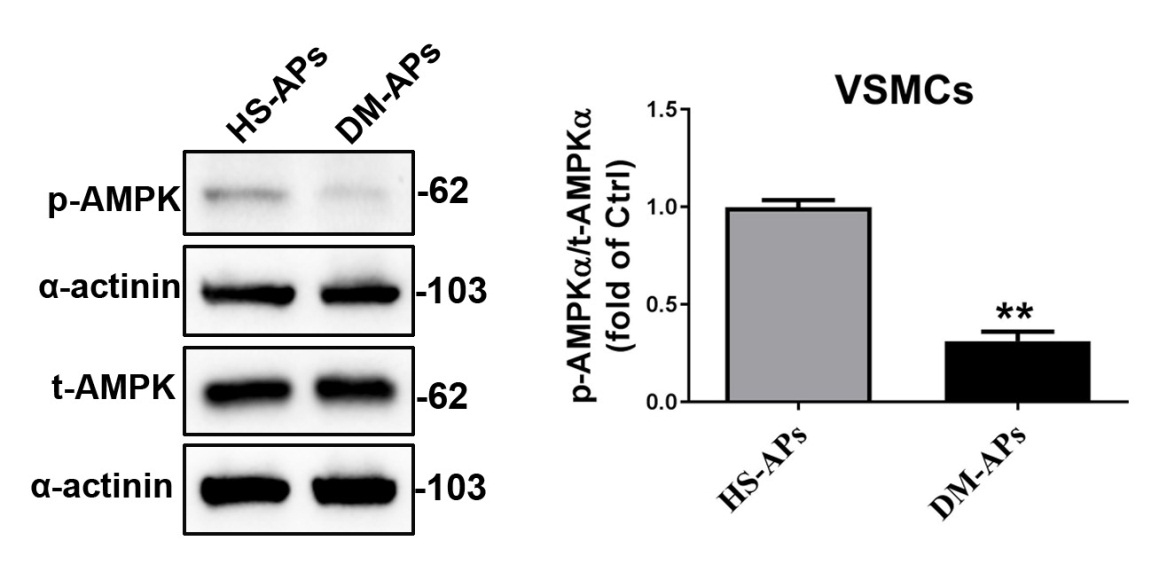


**Figure. S3. HS-APs activate the AMPKα signal pathway to suppress VSMC proliferation.** VSMCs were cocultured with the purified platelets from HS and DM patient. The phosphorylation ofAMPKαwas determined with western blot analysis in VSMCs with treatment of HS-APs and DM-APs. Data are presented as mean ± SD (n=3). **P < 0.01 vs. VSMCs with treatment of HS-APs.


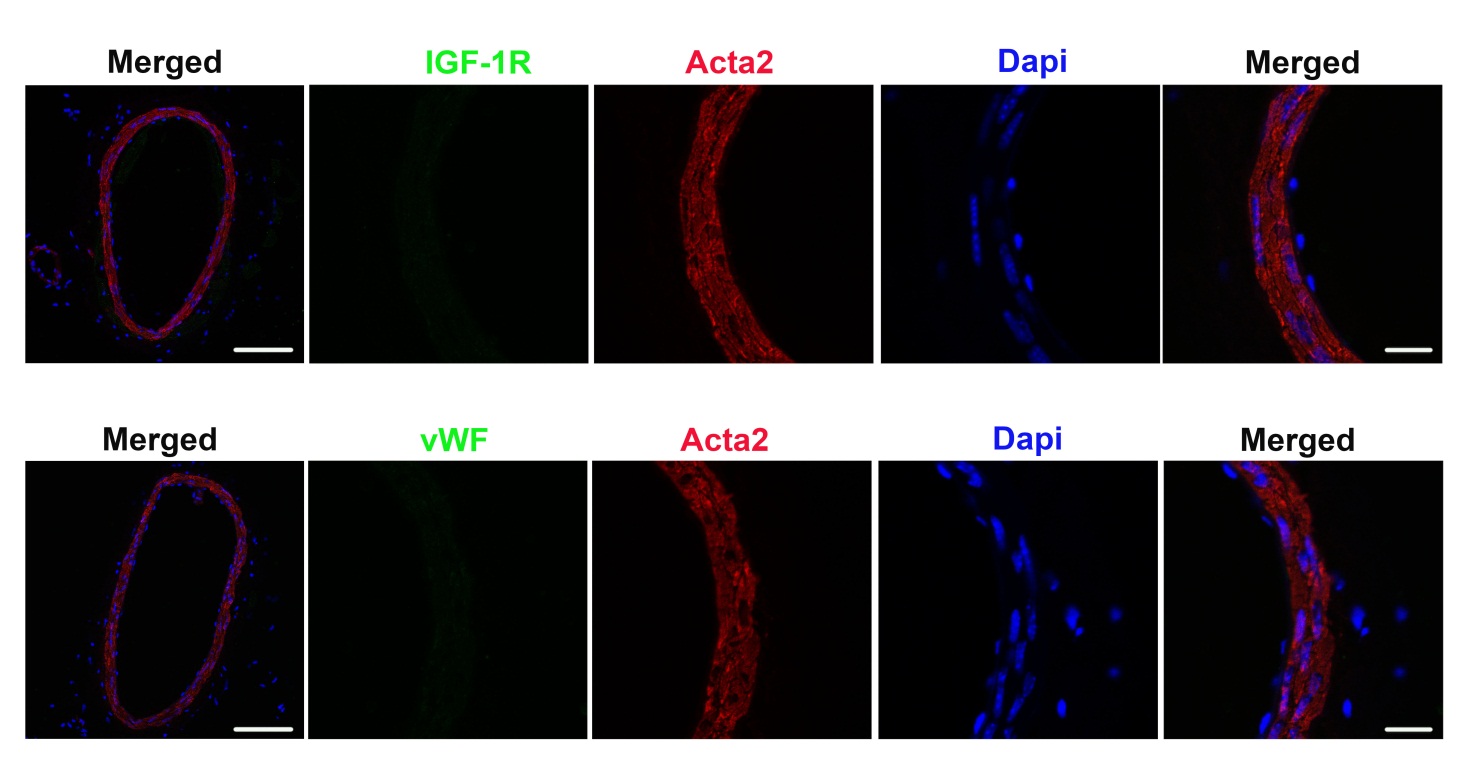


**Figure. S4. The non-immune immunoglobulin controls were showed to distinguish the specific staining.** Green, IGF-1R or vWF; red, Acta2 blue; Dapi nuclear staining in VSMCs. Scale bars: 20 μm, n = 3.


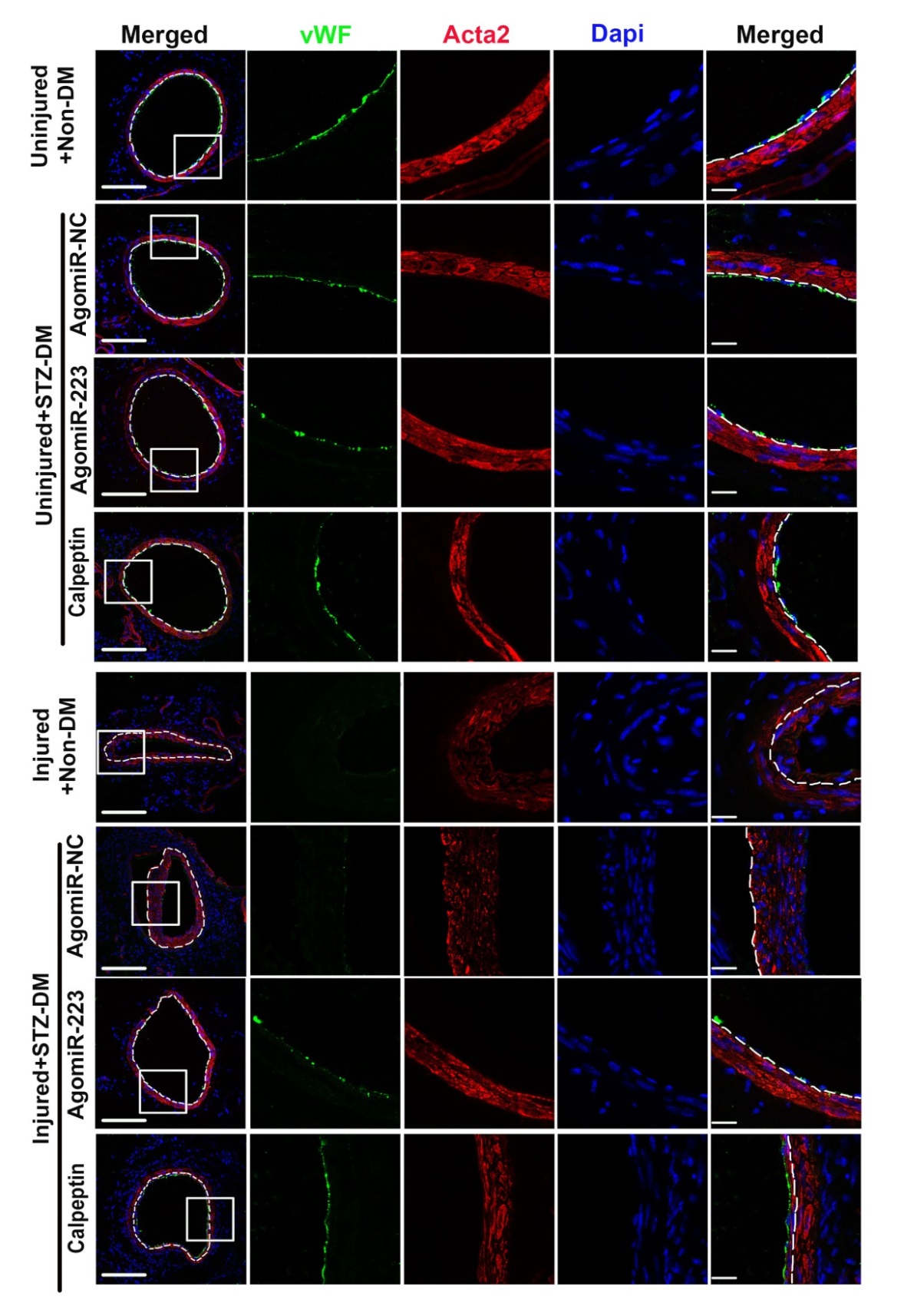


**Figure. S5. Inhibition of calpain alleviates endothelial coverage by restoring the expression of miR-223 in diabetic platelet.** Immunofluorescence analysis of vWF in uninjured or injured femoral arteries from Non-DM mice, and STZ-DM mice treated with AgomiR-NC or AgomiR-223 or calpeptin at 4 weeks after wire injury (n = 3). Green, vWF; red, Acta2 blue; Dapi nuclear staining in VSMCs. Scale bars: 20 μm.
