# Supplementary material for: Restoring the Platelet miR-223 by Calpain Inhibition Alleviates the Neointimal Hyperplasia in Diabetes
Source: Front Physiol. 2020 Jul 7;11:742. doi: 10.3389/fphys.2020.00742 (PMC7359912; doi:10.3389/fphys.2020.00742)
Supplement: Supplementary file 2 [file Data_Sheet_2.docx]

Supplementary Material

# Supplementary Tables

**Table 1. Primers for miRNA PCR**

| Primer | Forward Sequence | Reverse Sequence |
| --- | --- | --- |
| Has-miR-223 | GTTGCTCCTGTCAGTTTG TCAAA | TATGGTTGTTCACGACT CCTTCAC |
| mmu-mir-223 | GTTGCTCCTGTCAGTTTG TCAAA | TATGGTTGTTCACGACT CCTTCAC |
| U6 | ATTGGAACGATACAGAG AAGATT | GGAACGCTTCACGAATT TG |

**Table 2. The sequences for miRNA mimic**

| Gene | Sense (5'-3') | antisense (5'-3') |
| --- | --- | --- |
| Negative control | UUCUCCGAACGUGUCA  CGUTT | ACGUGACACGUUCG  GAGAATT |
| hsa-miR-223 mimics | UGUCAGUUUGUCAAAU  ACCCCA | GGGUAUUUGACAAA  CUGACAUU |

**Table 3. Clinical characteristic of the DM donors**

| Donors | **Age sex** | PLT (x10^9^/L) | HbA1c  (%)/ (mmol/mol) | Serum glucose (mmol/L) |
| --- | --- | --- | --- | --- |
| 1 | 9 years old  Female | 300 | 6.70 | 10.62 |
| 2 | 12 years old  Female | 306 | 7.90 | 6.24 |
| 3 | 12 years old  Male | 227 | 8.40 | 9.08 |
| 4 | 7 years old  Male | 297 | 6.50 | 12.27 |
| 5 | 11 years old  Female | 218 | 7.10 | 9.64 |
| 6 | 14 years old  Female | 256 | 8.0 | 20.68 |

Clinical characteristic of DM parameters (HbA1c >=6.5%) subjects. The morphological parameters: Platelet count, HbA1c and serum glucose were showed in DM subjects (n=6).
